# Supplementary material for: Ultraprocessed Products as Food Fortification Alternatives: A Critical Appraisal from Latin America
Source: Nutrients. 2022 Mar 29;14(7):1413. doi: 10.3390/nu14071413 (PMC9002678; doi:10.3390/nu14071413)
Supplement: Supplementary file 1 [file nutrients-14-01413-s001.zip › nutrients-1615095-supplementary.pdf]

Supplementary Material

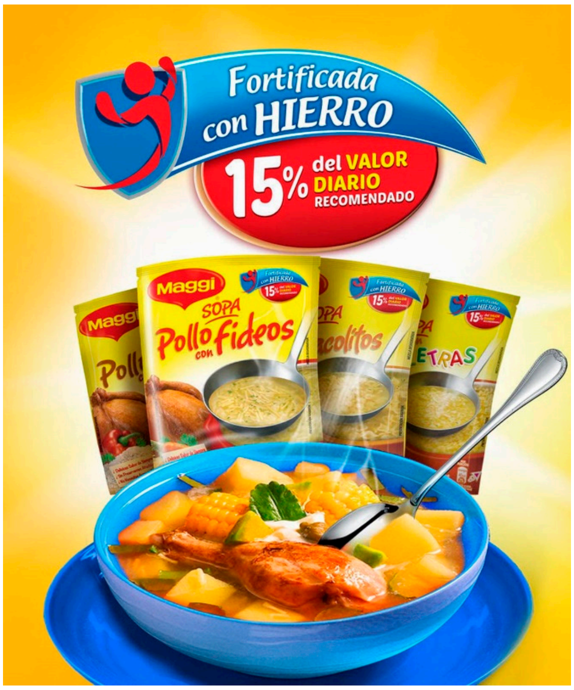

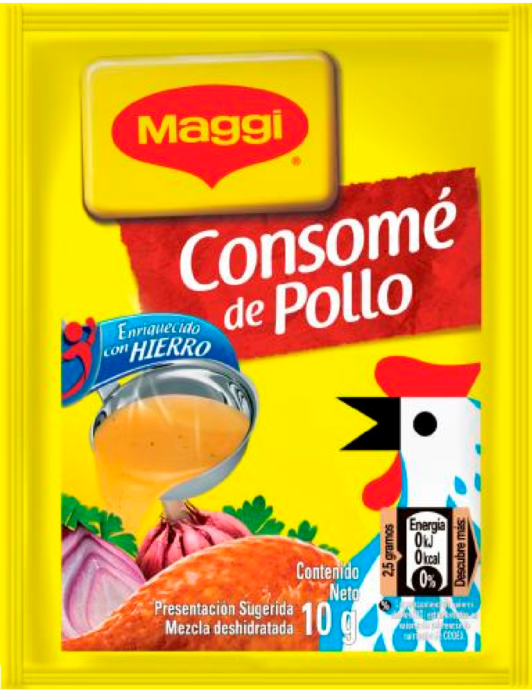

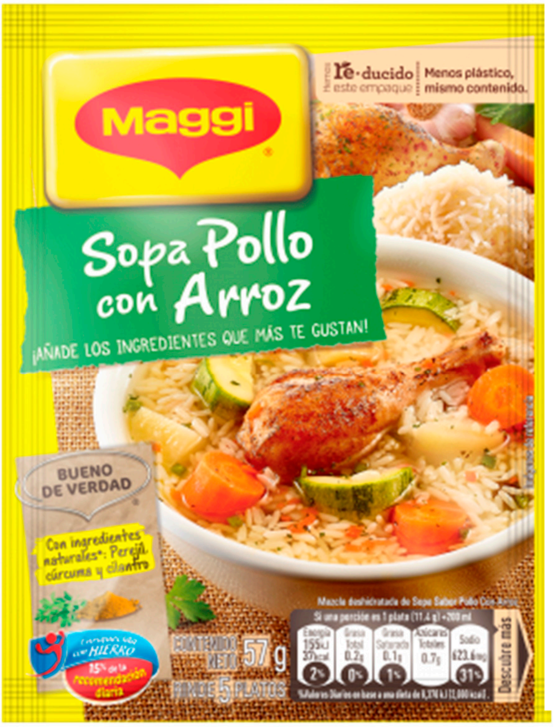

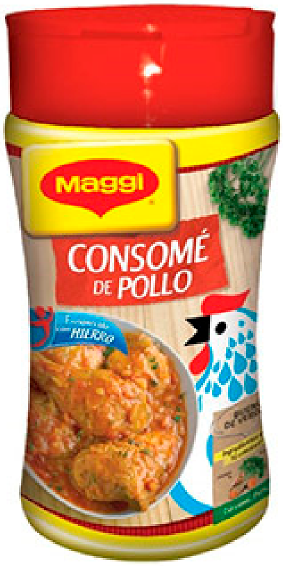

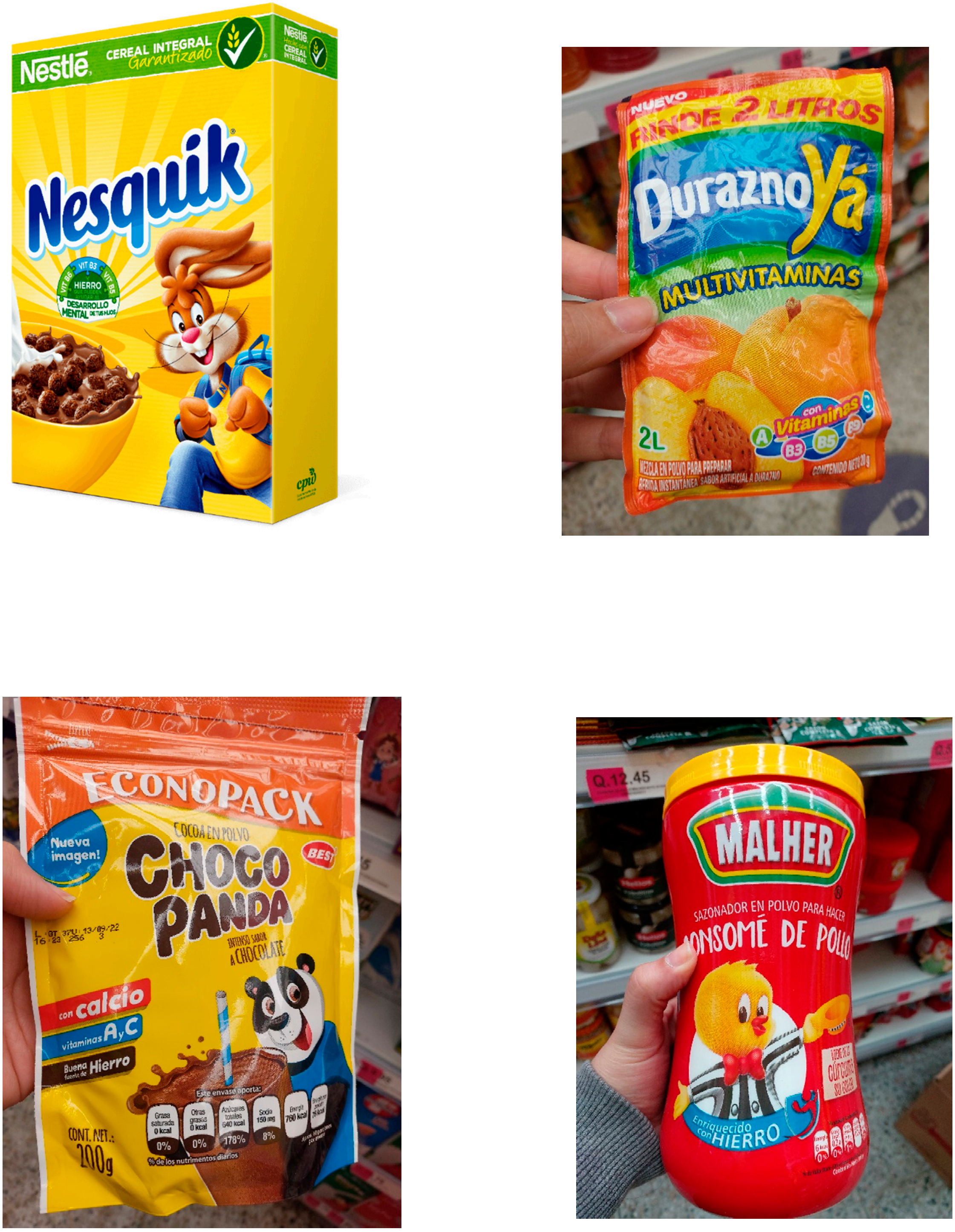

Figure S1. Examples of nutrient-related claims and marketing strategies of ultraprocessed products in Central American countries
